# Natural Cubic Spline Regression Modeling Followed by Dynamic Network Reconstruction for the Identification of Radiation-Sensitivity Gene Association Networks from Time-Course Transcriptome Data

**DOI:** 10.1371/journal.pone.0160791

**Published:** 2016-08-09

**Authors:** Agata Michna, Herbert Braselmann, Martin Selmansberger, Anne Dietz, Julia Hess, Maria Gomolka, Sabine Hornhardt, Nils Blüthgen, Horst Zitzelsberger, Kristian Unger

**Affiliations:** 1 Research Unit Radiation Cytogenetics, Helmholtz Zentrum München, German Research Center for Environmental Health GmbH, Neuherberg, Germany; 2 Clinical Cooperation Group "Personalized Radiotherapy in Head and Neck Cancer", Helmholtz Zentrum München, Neuherberg, Germany; 3 Department of Radiation Protection and Health, Federal Office for Radiation Protection, Neuherberg, Germany; 4 Institute of Pathology, Charité—Universitätsmedizin Berlin, Berlin, Germany; University of São Paulo, BRAZIL

## Abstract

Gene expression time-course experiments allow to study the dynamics of transcriptomic changes in cells exposed to different stimuli. However, most approaches for the reconstruction of gene association networks (GANs) do not propose prior-selection approaches tailored to time-course transcriptome data. Here, we present a workflow for the identification of GANs from time-course data using prior selection of genes differentially expressed over time identified by natural cubic spline regression modeling (NCSRM). The workflow comprises three major steps: 1) the identification of differentially expressed genes from time-course expression data by employing NCSRM, 2) the use of regularized dynamic partial correlation as implemented in GeneNet to infer GANs from differentially expressed genes and 3) the identification and functional characterization of the key nodes in the reconstructed networks. The approach was applied on a time-resolved transcriptome data set of radiation-perturbed cell culture models of non-tumor cells with normal and increased radiation sensitivity. NCSRM detected significantly more genes than another commonly used method for time-course transcriptome analysis (BETR). While most genes detected with BETR were also detected with NCSRM the false-detection rate of NCSRM was low (3%). The GANs reconstructed from genes detected with NCSRM showed a better overlap with the interactome network Reactome compared to GANs derived from BETR detected genes. After exposure to 1 Gy the normal sensitive cells showed only sparse response compared to cells with increased sensitivity, which exhibited a strong response mainly of genes related to the senescence pathway. After exposure to 10 Gy the response of the normal sensitive cells was mainly associated with senescence and that of cells with increased sensitivity with apoptosis. We discuss these results in a clinical context and underline the impact of senescence-associated pathways in acute radiation response of normal cells. The workflow of this novel approach is implemented in the open-source Bioconductor R-package splineTimeR.

## Introduction

In general terms, the expression of genes can be studied from a static or temporal point of view. Static microarray experiments allow measuring gene expression responses only at one single time point. Therefore, data obtained from those experiments can be considered as more or less randomly taken snapshots of the molecular phenotype of a cell. However, biological processes are dynamic and thus, the expression of a gene is a function of time [1]. To be able to understand and model the dynamic behavior and association of genes, it is important to study gene expression patterns over time.

However, compared to static microarray data, the analysis of time-course data introduces a number of new challenges. First, the experimental costs for the generation of data as well as the computational cost increases with the increase in the number of introduced time points. Second, hidden correlation caused by co-expression of genes makes the data linearly dependent [2]. Finally, one has to be aware of additional correlations existing between neighboring time points clearly revealed in published gene expression profiles [3].

Several different algorithms have been suggested to analyze gene time-course microarray data with regard to differential expression in two or more biological groups (e.g. exposed to radiation vs. non-exposed) [4–7]. Nevertheless solitary identification of differentially expressed genes does not help to determine the molecular mechanisms in the investigated biological groups. Therefore, it is not only important to know differentially expressed genes per se, but also how those genes interact and regulate each other in order to determine specifically deregulated molecular networks.

Currently, many different algorithms including cluster analysis [8–13] and supervised classification [14–16] are used to identify relationships between genes. However, both of these methods suffer from serious limitations. First, the timing information of the measurements is not incorporated and, therefore, the intrinsic temporal structure of the time-course data is neglected. Second, the available standard clustering and classification methods are not designed to measure statistical significance of the results based on a statistical hypothesis test. By nature of these methods, clusters or classes of genes with similar expression patterns will always be identified but they do not provide a measure of how reliable this information is. For this reason, we preferred usage of a dynamic network modeling approach that allows delineation of relationships between genes along with providing statistical significance for these relationships.

The aim of the present study was to identify and compare signaling pathways involved in the radiation responses of normal cells differing in their radiation sensitivity that could be used to modulate cell sensitivity to ionizing radiation. For this, we propose an approach that combines the detection of genes differentially expressed over time based on statistics determined by natural cubic spline regression (NCSRM) [17] followed by dynamic gene association network (GAN) reconstruction based on a regularized dynamic partial correlation as implemented in the GeneNet R-package [18].

Most exploratory gene expression studies focus only on the identification of differentially expressed genes by treating them as independent events and do not seek to study the interplay of identified genes. This makes it difficult to tell which genes are part of the interaction network causal of the studied phenotype and which are the most “important” with regard to the context of the investigation. The herein present approach combines the identification of differentially expressed genes and reconstruction of possible associations between them. Further analysis of identified GANs then allows hypothesizing which genes may play a crucial role in the investigated processes. This should markedly increase the likelihood to find meaningful results from an initial observation and help to understand the underlying molecular mechanisms. We applied our workflow on time-course transcriptome data of two normal and well-characterized lymphoblastoid cell lines with normal (20037–200) and increased radiation sensitivity (4060–200), in order to identify molecular mechanisms and potential key players responsible for different radiation responses [19, 20]. Our exploratory approach provides novel and informative insights in the biology of radiation sensitivity of non-tumor cells after exposure to ionizing radiation with regard to the identified signaling pathways and their key drivers. Moreover, we could demonstrate that spline regression in differential gene expression analysis for the purpose of prior selection in gene-association network reconstruction outperforms another commonly used existing approach for time-course gene expression analysis.

## Results

**Fig 1:**
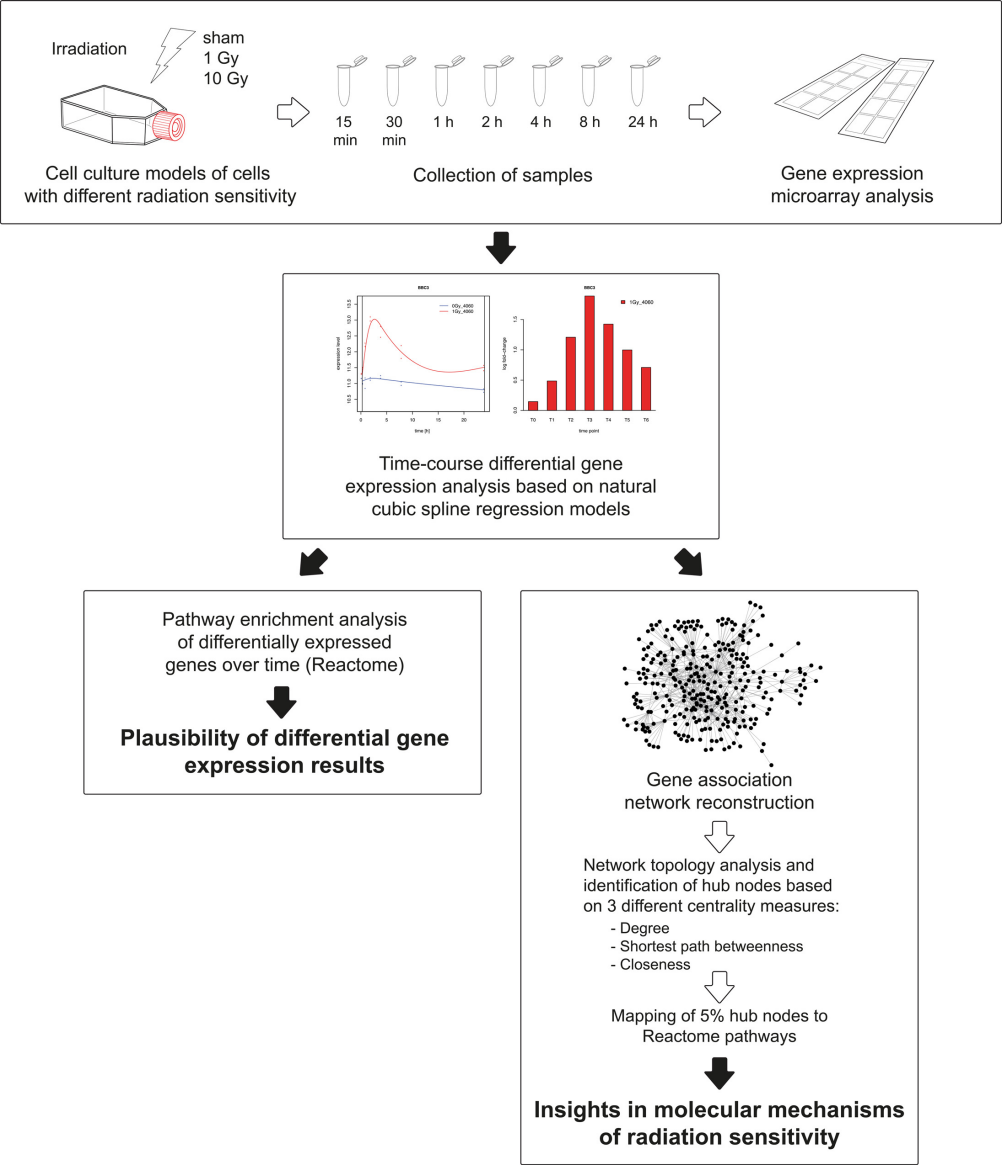
Schematic workflow of the analysis of gene expression time-course data. Samples were collected 0.25, 0.5, 1, 2, 4, 8 and 24 hours after sham or actual irradiation. Transcriptional profiling was performed using Agilent gene expression microarrays and comprises three major steps: the identification of differentially expressed genes from time-course expression data by employing a natural cubic spline regression model; the use of regularized dynamic partial correlation method to infer gene associations networks from differentially expressed genes and the topological identification and functional characterization of the key nodes in the reconstructed networks.

The schematic workflow of the presented novel approach for time-course gene expression data analysis is presented in Fig 1.

### Identification of ionizing radiation-responsive genes using NCSRM method

A fraction of the probes was removed due to low expression levels, with not detectable signal intensities as described in [21]. Table 1 shows the number of probes remained after quality filtering from the total number of 25220 unique probes representing HGNC annotated genes. Differential analysis was performed relative to the corresponding sham irradiated cells as a reference. In general, more genes were detected as differentially expressed in the cells with increased radiation sensitivity compared to cells with normal radiation sensitivity after each dose of gamma irradiation (Table 1). The most prominent difference was observed when comparing the responses after 1 Gy irradiation. In the cells with increased radiation sensitivity 2335 genes showed differential expression compared to only seven genes in cells with normal radiation sensitivity. We observed the same trend after irradiation with 10 Gy where the cells with increased sensitivity showed 6019 and the normal sensitive cells 3892 differentially expressed genes.

**Table 1:** Number of detected and differentially expressed genes for each dose and cell lines for NCSRM and BETR methods.

cell line and applied radiation dose	increased sensitivity (1 Gy vs 0 Gy)	Normal sensitivity (1 Gy vs 0 Gy)	increased sensitivity (10 Gy vs 0 Gy)	Normal sensitivity (10 Gy vs 0 Gy)
total number of detected probes after preprocessing	10388	11311	10330	11446
differentially expressed genes detected with **NCSRM**	2335	7	6019	3892
differentially expressed genes detected with **BETR**	923	12	3889	1256
intersection of differentially expressed genes resulting from both methods	855	4	3875	1233

### Pathway enrichment analysis of NCSRM identified genes

Pathway enrichment analysis was performed on differentially expressed genes to identify over-represented biological pathways. The analysis on genes identified with NCSRM revealed 634 and 964 significantly enriched pathways for the cells with increased radiation sensitivity after 1 Gy and 10 Gy irradiation dose, respectively and 758 pathways for the normal sensitive cell line after 10 Gy irradiation. For the seven differentially expressed genes (i.e. FDXR, BBC3, VWCE, PHLDA3, SCARF2, HIST1H4C, PCNA) of the cell line with normal radiation sensitivity after 1 Gy dose of irradiation we did not find any significantly enriched pathways. A summary of the pathway enrichment results can be found in S2 Table.

### Gene association network reconstruction

None of the edge probabilities calculated for the seven differentially expressed genes in the cell line with normal radiation sensitivity after 1 Gy irradiation exceeded the considered significance threshold and hence no network was obtained. For the remaining conditions we were able to obtain association networks as presented in Table 2. Obtained networks are provided as igraph R-objects in the supplementary data (S1 File). The graph densities for all resulting networks were in the same range as the density of the Reactome interaction network (Table 2).

**Table 2:** Number of genes subjected to GAN reconstruction and properties of resulted GANs.

method	NCSRM				BETR			
cell line and applied radiation dose	Increased sensitivity (1 Gy)	normal sensitivity (1 Gy)	Increased sensitivity (10 Gy)	normal sensitivity (10 Gy)	Increased sensitivity (1 Gy)	normal sensitivity (1 Gy)	Increased sensitivity (10 Gy)	normal sensitivity (10 Gy)
number of genes taken for network reconstruction	2335	7	6019	3892	923	12	3889	1256
number of nodes remained in the network	1140	-	3483	2735	336	-	2299	773
number of edges in the network	12198	-	114629	84695	3268	-	126378	16862
network density	0.00939	-	0.00945	0.01133	0.02903	-	0.02392	0.02826
density of the Reactome interaction network	0.00536							

Gene association network reconstructions were performed using the GeneNet method [18]. Association between two genes was considered as significant if posterior edge probability was equal or greater than 0.95. Densities of the reconstructed networks were compared with the density of the Reactome interaction network in order to assess their complexity.

### Identification and functional characterization of the most important genes in the reconstructed association networks

The combined topological centrality measure was used to characterize the biological importance of nodes (genes) in the reconstructed association networks. The 5% of the highest ranked genes listed in supplementary S3 Table were mapped to Reactome pathways in order to further evaluate their biological roles. The top 10 most relevant pathways according to the FDR values are shown in Table 3. For the cell line with increased radiation sensitivity after irradiation with 1 Gy and for the normal sensitive cell line after 10 Gy the induction of pathways associated with senescence response was detected. For the cell line with increased radiation sensitivity after 10 Gy of irradiation we mostly observed pathways associated with apoptosis. All pathways are listed in supplementary S4 Table.

**Table 3:** Comparison of NCSRM and BETR methods with respect to the top 10 pathways after mapping of 5% highest ranked genes from the reconstructed gene association networks.

with NCSRM method			with BETR method		
increased sensitivity (1 Gy)	increased sensitivity (10 Gy)	normal sensitivity (10 Gy)	increased sensitivity (1 Gy)	increased sensitivity (10 Gy)	normal sensitivity (10 Gy)
Signal Transduction	Signal Transduction	Generic Transcription Pathway	DNA Damage/Telomere Stress Induced Senescence^a^	Activation of BH3-only proteins^b^	DNA Damage/Telomere Stress Induced Senescence^a^
Cellular Senescence^a^	Activation of BH3-only proteins^b^	DNA Damage/Telomere Stress Induced Senescence^a^	Senescence-Associated Secretory Phenotype (SASP)^a^	Activation of PUMA and translocation to mitochondria^b^	Generic Transcription Pathway
DNA Damage/Telomere Stress Induced Senescence^a^	Activation of PUMA and translocation to mitochondria^b^	Immune System	Signal Transduction	Cytokine Signaling in Immune system	Cellular Senescence^a^
Formation of Senescence-Associated Heterochromatin Foci (SAHF)^a^	Fatty acid, triacylglycerol, and ketone body metabolism	Gene Expression	Activated PKN1 stimulates transcription of AR (androgen receptor) regulated genes KLK2 and KLK3	Immune System	Gene Expression
Cellular responses to stress	Metabolism	Inositol phosphate metabolism	Cell Cycle Checkpoints	Intrinsic Pathway for Apoptosis^b^	Meiotic recombination
RAF-independent MAPK1/3 activation	Metabolism of proteins	IRF3-mediated induction of type I IFN	Cellular Senescence^a^	Signal Transduction	Signal Transduction
Signaling by ERBB4	PPARA activates gene expression	Cellular Senescence^a^	DNA methylation	Gene Expression	Cell Cycle
DAP12 interactions	Regulation of lipid metabolism by Peroxisome proliferator-activated receptor alpha (PPARalpha)	Formation of Senescence-Associated Heterochromatin Foci (SAHF)^a^	Packaging Of Telomere Ends	BH3-only proteins associate with and inactivate anti-apoptotic BCL-2 members^b^	Transcriptional activation of cell cycle inhibitor p21
PRC2 methylates histones and DNA	Activation of gene expression by SREBF (SREBP)	STING mediated induction of host immune responses	RNA Polymerase I Promoter Opening	Activation of the mRNA upon binding of the cap-binding complex and eIFs, and subsequent binding to 43S	Transcriptional activation of p53 responsive genes
Apoptotic execution phase^b^	BH3-only proteins associate with and inactivate anti-apoptotic BCL-2 members^b^	Metabolism	SIRT1 negatively regulates rRNA Expression	Endosomal/Vacuolar pathway	Senescence-Associated Secretory Phenotype (SASP)^a^

^a^Pathways associated with senescence responses.

^b^Pathways associated with apoptotic processes.

### False detected differentially expressed genes between technical replicates

In order to assess the false positive rate, the spline regression based differential analyses between technical replicates of each treatment conditions and cell lines were performed. Here, we can state that the null-hypothesis of no differential expression is true for all genes. Then the q*-level of 0.05 for Benjamini-Hochberg method controls also the FWER at alpha-level equal to 0.05 (type I error) [22]. For all compared technical replicates not more than 3% rejections of null hypothesis were detected, which is in good accordance to the expected or nominal type I error.

### Evaluation of spline regression model in comparison to BETR method

Table 1 compares the numbers of differentially expressed genes obtained from both methods applied on the same gene expression data set and FDR thresholds. For almost all treatment conditions the BETR method detected less differentially expressed genes in comparison to NCSRM. Only for the normal cell line after irradiation with 1 Gy BETR identified 12 genes whereas NCSRM identified only 7 genes. As a consequence of the lower numbers of detected differentially expressed genes with BETR, the obtained networks are smaller than those obtained after spline regression. The detailed comparison results including numbers of detected differentially expressed genes and the sizes of reconstructed association networks are presented in the Table 2. The lists of differentially expressed genes obtained with the two methods are shown in supplementary S1 Table. The top 10 pathways to which the 5% of the most important genes in the reconstructed association networks where mapped to are shown in Table 3. With NCSRM we were not only able to detect almost all genes that were detected also by BETR (Table 1), but also an additional set of genes resulting in almost twice the number of genes compared to BETR. Nevertheless, the top 5% hub genes of the networks derived from the differentially expressed genes defined by BETR were associated with similar biological processes as those from the spline differential expression analysis derived networks. The numbers and names of overlapping hub genes in the GANs are presented in Table 4 and in supplementary S3 Table, respectively.

**Table 4:** Comparison of hub genes in networks resulting from different methods.

cell line and applied radiation dose	increased sensitivity (1 Gy)	increased sensitivity (10 Gy)	Normal sensitivity (10 Gy)
5% hub genes in the NCSRM resulting network in numbers	57	174	137
5% hub genes in the BETR resulting network in numbers	17	115	39
number of common hub genes resulting from both methods	9	111	31

### Evaluation of reconstructed networks

The evaluation of the two networks derived after 1 Gy irradiation of the cell line with increased sensitivity showed that the network reconstructed with the differentially expressed genes determined using BETR did not contain significantly more common edges than random networks (p = 0.529), whereas the network reconstructed with the differentially expressed genes determined by NCSRM did (p = 0.048). The networks derived after 10 Gy irradiation of the cell line with increased sensitivity and 10 Gy irradiation of the normal sensitive cell line contained significantly more edges that were common with the Reactome network compared to random networks for both methods.

## Discussion

The success of tumor radiation therapy predominantly depends on the total applied radiation dose, but also on the tolerance of the tumor surrounding normal tissues to radiation. Toxicity towards radiation, which greatly varies on an individual level due to inherited susceptibility, is one of the most important limiting factors for dose escalation in radiooncology treatment [23, 24]. To account for radiation sensitivity of normal tissue in personalized treatment approaches the underlying molecular mechanisms need to be thoroughly understood in order to identify molecular targets for the modulation of radiation sensitivity and molecular markers for the stratification of patients with different intrinsic radiation sensitivity. In the present study we identified significantly differentially expressed genes over time between the radiation-treated group and the control group to be used as prior genes for GAN reconstruction. Two doses of gamma irradiation were used to characterize the differences in radiation response of the two lymphoblastoid cell lines with known differences in radiation sensitivity. The dose of 10 Gy was selected following the fact that the same dose has been applied in a previous research project examining the radiation sensitivity of the same lymphoblastoid cell lines analyzed in the study at hand [20]. The dose of 1 Gy reflects the dose that is delivered as part of the so called “low-dose bath” to the tumor-surrounding tissue during the radiotherapy of the tumors [25].

Here, we conducted time-resolved transcriptome analysis of radiation-perturbed cell culture models of non-tumor cells with normal and with increased radiation sensitivity in order to work out the molecular phenotype of radiation sensitivity in normal cells. Moreover, we present an innovative approach for the identification of GANs from time-course perturbation transcriptome data. The approach comprises three major steps: 1) the identification of differentially expressed genes from time-course gene expression data by employing a natural cubic spline regression model (NCSRM); 2) the use of a regularized dynamic partial correlation method to infer gene associations network from differentially expressed genes; 3) the identification and functional characterization of the key nodes (hubs) in the reconstructed gene dependency network (Fig 1).

Our proposed method for the detection of differentially expressed genes over time is based on NCSRM with a small number of basis functions. A relatively low number of basis functions generally results in a good fit of data and, at the same time, reduces the complexity of the fitted models. Treating time in the model as a continuous variable, a non-linear behavior of gene expressions was approximated by spline curves fitted to the experimental time-course data. Considering temporal changes in gene expression as continuous curves and not as single time points greatly decreases the dimensionality of the data and thereby decreases computational cost. In addition, the proposed NCRSM does not require identical sampling time points for the compared treatment conditions. Furthermore, no biological replicates are needed. Therefore, the method is applicable to data generated according to a tailored time-course differential expression study design and to data that were not specifically generated for time-course differential expression analysis, e.g. existing/previously generated data from clinical samples. Thus, the adaption of the method to differential expression analysis comprises the potential to reanalyze existing data, address new questions *in silico* and thereby potentially add new or additional value to existing data. Incomplete time-course data, e.g. due to the exclusion of samples for technical reasons, that often create major problems for the estimation of the model, are also suitable for fitting the spline regression model as long as enough data points remain in the data set. This is especially valuable when data on certain time points, derived from a very limited sample source, have been excluded from a time-course data set and cannot be repeatedly generated.

Since gene expression is not only dynamic in the treatment group but also in the control group, the inclusion of the time-course control data greatly improves the ability to detect truly differentially expressed genes, as the gene expression values are not referred to a single time point with static gene expression levels only. Comparing a treatment group to time point zero does not provide a proper control over the entire time-course, although it is widely practiced [26–28]. The proposed workflow is implemented in an open-source R-package splineTimeR and is available through Bioconductor (https://www.bioconductor.org).

Amongst a panel, the two lymphoblastoid cell lines that were different with regard to radiation sensitivity after irradiation with 10 Gy [20], also responded differently with regard to the quantity of differentially expressed genes. Interestingly, cells with normal radiation sensitivity barely responded to 1 Gy irradiation at the transcriptome level. Only seven genes (FDXR, BBC3, VWCE, PHLDA3, SCARF2, HIST1H4C, PCNA) were identified as differentially expressed, whereas for the cell line with increased sensitivity 2335 differentially expressed genes were detected after exposure to the same dose. A similar behavior was observed for those two cell lines after irradiation with 10 Gy. We detected 6019 and 3892 genes as differentially expressed in the sensitive and normal cell lines, respectively (Table 2). Those results are in a good agreement with the previous proteomic study where more differentially expressed proteins were detected for the same sensitive cell line compare to the cell line with normal radiation sensitivity 24 hours after irradiation with 10 Gy [29]. Thus, for both applied doses, the radiation sensitive cells exhibited much more pronounced transcriptional response compared to the cells with normal radiation sensitivity and thereby underlines the expected radiation response of those two cell lines.

Concerning qualitative differences in the transcriptomic response of normal sensitive cells and cells with increased sensitivity after treatment with 1 Gy and 10 Gy pathway enrichment analysis was performed. Differentially expressed genes identified for all considered treatment conditions except for the normal sensitive cells after exposure to 1 Gy radiation showed statistically significant enrichment of pathways. Most of which were in agreement with known radiation responses such as DNA repair, cell cycle regulation, oxidative stress response or pathways related to apoptosis (S2 Table) [30–32]. Therefore, the pathway enrichment analysis results suggest plausibility of generated data and, more importantly, underline the meaningfulness of our suggested approach based on cubic spline regression for differential gene expression analysis of time-course data. However, differential expression analysis alone followed by pathway enrichment analysis does not provide any mechanistic insights. For this reason we performed GAN reconstruction using identified differentially expressed genes. Based on the assumption that the expression levels of functionally related genes are highly correlated, partial correlation was used for GAN reconstruction. In simple correlation, the strength of the linear relationship between two genes is measured, without taking into account that those genes may be actually influenced by other genes. Partial correlation eliminates the influence of other genes when one particular relationship between pair of genes is considered. Network reconstruction was performed separately for the cell line with increased radiation sensitivity after 1 Gy and 10 Gy and for the cell line with normal radiation sensitivity after 10 Gy of radiation dose. Due to the sparseness of the set of genes differentially expressed after irradiation of the normal-sensitive cell line with 1 Gy, no GAN was obtained.

Subsequently, we identified the network hubs (i.e. most important genes) of the GANs by combining three network centrality measures: degree, closeness and shortest path betweenness [33]. Combining different centrality measures is a widely used approach to identify nodes that are likely to control the network [34]. Also, this approach allows identification of nodes that are connected to the central nodes at the same time which can be informative for the interpretation of the whole GAN or single modules making up the network [33, 34].

### Identification of key pathways associated with radiation sensitivity

In order to get functional insights into the reconstructed GANs the 5% top important nodes were identified after a ranking with the combined centrality measure and mapped to the pathways from the interactome database Reactome [35]. The obtained results revealed different pathways considered as the most important in cells with different radiation sensitivity after different doses of ionizing radiation. For the radiation sensitive cell line 4060–200 and 1 Gy irradiation, we mainly detected pathways associated with senescence (Table 3).

A different outcome was observed after irradiation with 10 Gy. For the radiation sensitive cells three out of the ten top pathways were linked to apoptotic processes with the genes BBC3, BCL2, TP53 as key players, whereas for the normal sensitive cell line we mainly observed the induction of senescence related pathways. This indicates that different doses are necessary to induce a similar response in the two cell lines. The activation of senescence genes is a damage response mechanism, which stably arrests proliferating cells and protects them from apoptotic cell death [36]. Together with the senescence pathway we observed increased levels of chemokine, cytokine and interleukin genes that are known to activate an immune response and signal transduction pathways in response to irradiation.

Although the senescence-associated pathways were not seen as the most important ones for the treatment condition 10 Gy/increased sensitivity, they were significantly enriched in the GANs of the three conditions 1 Gy/increased sensitivity, 10 Gy/ increased sensitivity and 10 Gy/normal sensitivity. All differentially expressed genes that related to senescence-associated pathways are shown in supplementary S5 Table. The observation that cells with increased radiation sensitivity compared to cells with normal sensitivity, become senescent after exposure to doses in the range of 1 Gy, rises the question whether this has a positive or negative influence on the tumor therapy. On the one hand side, senescent cell may secret the so-called SASP (“senescence-associated secretory phenotype“) factors, including growth factors, chemokines and cytokines, which participate in intercellular signaling leading to the attraction of immune cells to the tumor location that, in turn, eliminate the tumor cells and, thereby, positively contribute to the tumor therapy [37, 38]. On the other hand side, senescent cells and the SASP are reported to promote proliferation, survival, invasion and migration of neighboring cells by the release of pro-inflammatory cytokines leading to sustained inflammation [36]. In this way senescence cells can damage their local environment and stimulate angiogenesis and tumor progression [39, 40]. Besides, there are some evidences that the induction of senescence in surrounding normal tissue may lead to an increased radio-tolerance or even radioresistance of the tumor and is, therefore, not desirable and negatively influences the tumor radiotherapy [41]. Thus, it might be beneficial to block senescence in order to prevent the radio-hyposensibilization of tumor cells. Therefore, we suggest a detailed investigation of the consequences of senescent non-tumor cells with the aim to improve the radiotherapy of tumors in radiosensitive patients.

### Identification of senescence associated genes involved in cell radiation responses

CDKN1A gene was identified as one of the most important key players linked to the identified senescence associated pathways for both 1 Gy/sensitive and 10 Gy/normal treatment conditions. For both conditions the expression of the CDKN1A was up-regulated for all considered time points. CDKN1A is a well-known damage response gene for which aberrant transcriptional response has been associated with abnormal sensitivity to ionizing radiation [42, 43]. The study by Badie et al. (2008) has shown that a subgroup of breast cancer patients, who developed severe reactions to radiation therapy, could be identified by aberrant overexpression of CDKN1 in peripheral blood lymphocytes [43].

LMNB1 is another genes we identified as a response hub gene after irradiation of sensitive cell line with 1 Gy radiation dose that is associated with senescence. Although the LMNB1 gene was not identified as hub gene in the GAN of the 10 Gy/normal treatment condition, it was still differentially expressed. For both treatment conditions we observed significant downregulation of this gene 24 hours after irradiation. Shah et al (2013) has suggested that downregulation of LMNB1 in senescence is a key trigger of chromatin changes affecting gene expression [44]. In fact also in our data we observed strong downregulation of a group of histone genes associated with senescence (S5 Table) for the treatment conditions 1 Gy/increased sensitivity and 10 Gy/normal sensitivity. Furthermore, Lee et al. (2012) has shown that histone protein modification may have an impact on the radiation sensitivity of a tissue [45]. Moreover, evidence has been provided that mutation of LMNA can cause increased sensitivity to ionizing radiation [46], however, to our knowledge there are no data showing the role of LMNB gene in the context of radiation sensitivity.

Another potential therapeutic candidate associated with senescence that was identified for the 10 Gy/normal sensitivity treatment condition was MRE11A for which cell culture data suggest that treatment of cells with Mre11 siRNA increases radiation sensitivity and reduces heat-induced radiosensitization [47, 48]. However, the clinical applicability of MRE11, has not been confirmed [49].

### Assessment of the false positive rate and validation of the NCSRM method

The spline regression based differential analyses between technical replicates were performed in order to estimate the extent of random fluctuations of gene expression values. The detected 3% rejections of the overall null hypothesis of no differential gene expression are in accordance with the alpha-level of 5% of the familywise error rate (FWER) and can be considered as false positives. On the other hand, it shows that type I error, due to technical variation, is covered by the model and test assumptions (moderated F-test, [50]) so that it was not necessary to include an extra parameter for technical replicates into the model.

In order to validate the previously mentioned biological results using NCSRM, we performed the differential expression analysis with another established method for time-course data analysis called BETR (Bayesian Estimation of Temporal Regulation) [6]. The number of genes detected by BETR was considerably lower compared to NCRSM (Table 1), however the majority of which were also detected with NCSRM (S1 Table). This is in line with the calculations on the false positive rates that have been conducted on the simulated data presented in the BETR study. In an analysis of the simulated data set, 65% of truly differentially expressed genes have been identified after accepting a false positive rate of 5% [6]. This means that a substantial proportion of differentially expressed genes remained undetected, which is likely to be also the case for the herein analyzed data with BETR. Although the numbers of differentially expressed genes and genes remained in the reconstructed networks greatly differ (Table 1), the qualitative results are well comparable (Table 3). For all treatment conditions where for which we were able to reconstruct GANs, we observed a great overlap of pathways where the 5% of hub genes were mapped to (Table 3). The detection of a higher number of differentially expressed genes with NCSRM resulted in larger GANs with additional information compared to the smaller GANs that were reconstructed on the basis of genes detected with BETR. This is underlined by the results of the conducted evaluation of GANs. Except one network based on the differentially expressed genes using BETR, all investigated networks consist significantly more common edges with the Reactome reference network compared to random networks with identical network topology and genes. This shows that the additionally detected genes with NCSRM add additional information rather than adding false positives or noise to the set of differentially expressed genes. Moreover the spline regression method is much more flexible and allows for more freedom during the data collection process. As already mentioned, NCSRM does not require the same sampling time for treated and control groups and can easily deal with incomplete data, whereas BETR method is not able to overcome or bypass those limitations. Thus, NCSRM is very robust against the frequently occurring shortcomings in study design and subsequent data generation occurring in life sciences.

### Conclusion

Prospectively, we suggest and plan a detailed *in silico* and *in vitro* analysis of the interactions in the proposed gene association networks in order to add meaningful knowledge to the mechanism of radiosensitivity at the experimental level. This novel knowledge has the potential to improve cancer radiation therapy by preventing or lowering the acute responses of normal cells resulting from radiation therapy. The results add novel information to the understanding of mechanisms that are involved in the radiation response of human cells, with the potential to improve tumor radiotherapy. Besides, the presented workflow is not limited to presented study only, but may be applied in other special fields with different biological questions to be addressed.

The software is provided as R-package “splineTimeR” and freely available via the Bioconductor project at http://www.bioconductor.org.

## Material and Methods

### Cell culture

Experiments were conducted with two monoclonal lymphoblastoid Epstein-Barr virus-immortalized cell lines (LCL) obtained from young lung cancer patients of the LUCY study (**LU**ng **C**ancer in **Y**oung) that differ in radiosensitivity, as tested with Trypan Blue and WST-1 assays [19, 20]. The non-cancer cell lines LCL 4060–200 with increased radiation sensitivity and LCL 20037–200 with normal radiation sensitivity were cultured at 37°C/5% CO_2_ in RPMI 1640 medium (Biochrom) supplemented with 10% fetal calf serum (FCS; PAA). Mycoplasma contamination was routinely tested using luminescence-based assays (MycoAlert, Lonza).

### Irradiation and sample preparation

The cells were seeded in 75 cm^2^ flasks at a concentration of 0.5 x 10^6^ cells/ml in a total volume of 60 ml. Exponentially growing cells were irradiated with sham, 1 Gy and 10 Gy of gamma-irradiation (^137^Cs-source HWM-D 2000, Markdorf, Germany) at a dose rate of 0.49 Gy/min. Samples were collected 0.25, 0.5, 1, 2, 4, 8 and 24 hours after sham or actual irradiation. Between the time of collection cells were kept in the incubator. Collected cells were washed with PBS and frozen at -80°C. Total RNA was isolated from frozen cell pellets obtained from two independent experiments using the AllPrep DNA/RNA/miRNA Universal Kit (Qiagen) including an DNase digestion step, according to the manufacturer's protocol. The concentration of RNA was quantified with a Qubit 2.0 Fluorometer (Life Technologies), and integrity was determined using a Bioanalyzer 2100 (Agilent Technologies). RNA samples with a RNA integrity number (RIN) greater than 7 indicated sufficient quality to be used in subsequent RNA microarray analysis.

### Gene expression profiling

Transcriptional profiling was performed using SurePrint G3 Human Gene Expression 8x60k V2 microarrays (Agilent Technologies, AMADID 39494) according to the manufacturer’s protocol. 75 ng of total RNA was used in labeling using the Low Input Quick Amp Labeling Kit (one-color, Agilent Technologies). Raw gene expression data were extracted as text files with the Feature Extraction software 11.0.1.1 (Agilent Technologies). The expression microarray data were uploaded to ArrayExpress (www.ebi.ac.uk/arrayexpress/) and the data set is available under the accession number E-MTAB-4829. All data analysis was conducted using the R statistical platform (version 3.2.2, www.r-project.org) [51]. Data quality assessment, filtering, preprocessing, normalization, batch correction based on nucleic acid labeling batches and data analyses were carried out with the Bioconductor R-packages limma, Agi4x44PreProcess and the ComBat function of the sva R-package [4, 21, 52]. All quality control, filtering, preprocessing and normalization thresholds were set to the same values as suggested in Agi4x44PreProcess R-package user guide [21]. Only HGNC annotated genes were used in the analysis. For multiple microarray probes representing the same gene the optimal probe was selected according to the Megablast score of probe sequences against the human reference sequence (http://www.ncbi.nlm.nih.gov/refseq/) [53]. If the resulted score was equal for two or more probes, the probe with the lowest differential gene expression FDR value was kept for further analyses since only one expression value per gene was allowed in subsequent GAN reconstruction analysis.

### Spline regression model for two-way experimental design

A natural cubic spline regression model (NCSRM) with three degrees of freedom for an experimental two-way design with one treatment factor and time as a continuous variable was fitted to the experimental time-course data. The mathematical model is defined by the following eq (1):

$$y=y(t,x)=b_{0}+b_{1}B_{1}(t-t_{0})+b_{2}B_{2}(t-t_{0})+\ldots +b_{m}B_{m}(t-t_{0})+x(d_{0}+d_{1}B_{1}(t-t_{0})+d_{2}B_{2}(t-t_{0})+\ldots +d_{m}B_{m}(t-t_{0}))$$

where b_0_, b_1_, …, b_m_ are the spline coefficients in the control group and d_0_, d_1_, …, d_m_ are differential spline coefficients between the control and the irradiated group. B_1_(t-t_0_), B_2_(t-t_0_), …, B_m_(t-t_0_) are the spline base functions and t_0_ is the time of the first measurement. For x = 0, y = y_control_ and for x = 1, y = y_irradiated_. For three degrees of freedom (df = 3), m = 3.

Depending on the number of degrees of freedom, two boundary knots and df-1 interior knots are specified. The interior knots were chosen at values corresponding to equally sized quantiles of the sampling time from both compared groups. For example, for df = 3 interior knots correspond to the 0.33- and 0.66-quantiles. The spline function is cubic on each defined by knots intervals, continuous at each knot and has continuous derivatives of first and second orders.

### Time-course differential gene expression analysis

**Fig 2:**
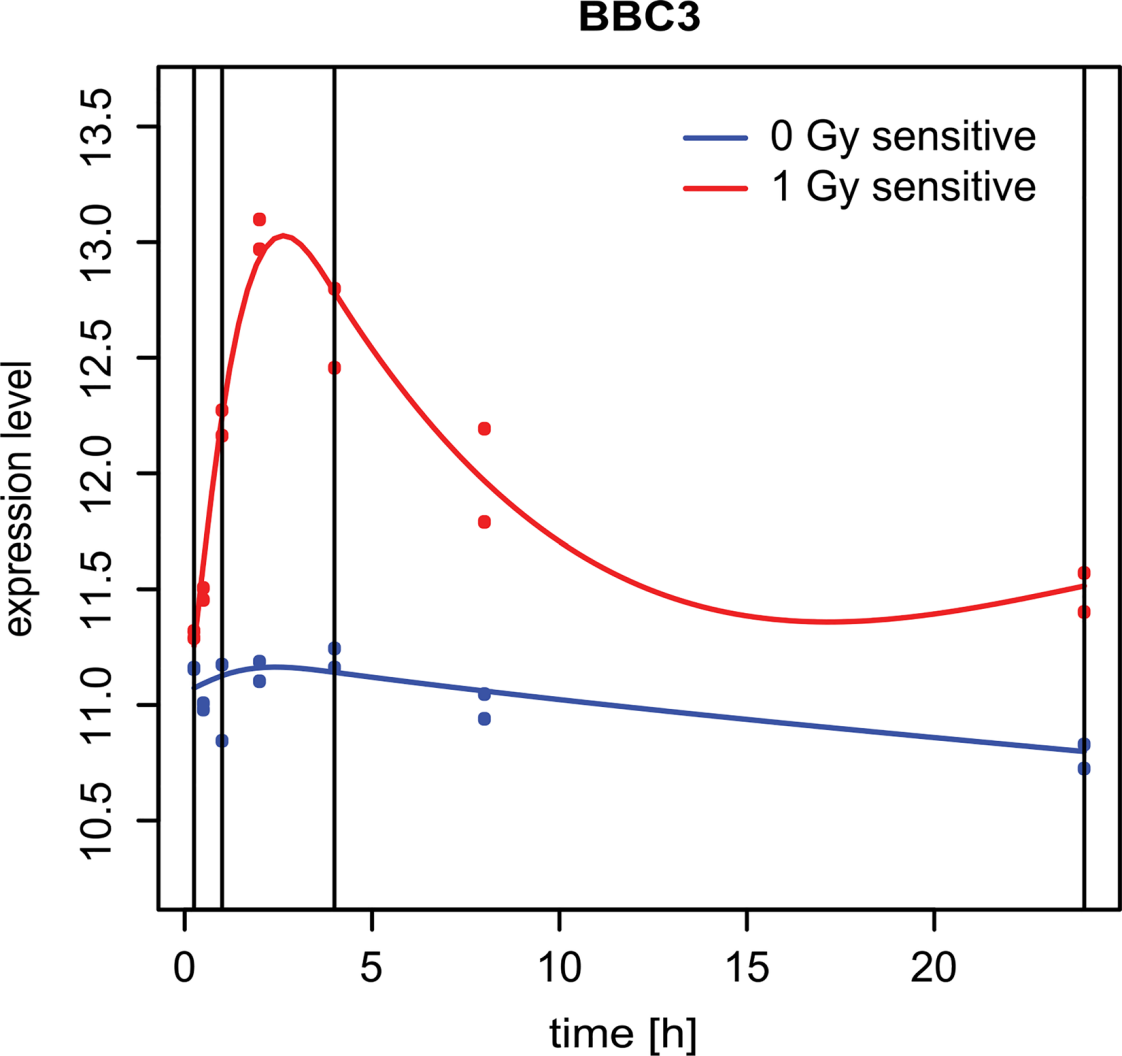
Example of fitted spline regression models. The plot shows spline regression models fitted to the measured time-course expression data of an arbitrary chosen gene (BBC3). The blue line represents the fitted model for the control (0 Gy) and read line that for the irradiated group (1 Gy). Blue and red dots represent the measured expression levels of the biological replicates. Vertical lines represent the endpoints and interior knots correspond to the 0.33- and 0.66-quantiles.

The time-course differential gene expression analyses were conducted between irradiated and control cells (sham-irradiated). Analyses were performed on the normalized gene expression data using NCSRM with three degrees of freedom. The splines were fitted to the real time-course expression data for each gene separately according to eq (1). The example of spline regression model fitted to the measured time-course data for one selected gene is shown on the Fig 2.

Time dependent differential expression of a gene between the irradiated and corresponding control cells was determined by the application of empirical Bayes moderated F-statistics [50] on the differential coefficients values in eq (1). In order to account for the multiple-testing error, corresponding p-values were adjusted by the Benjamini-Hochberg method for false discovery [22]. Genes with an adjusted p-value (FDR, false discovery rate) lower than 0.05 were considered as differentially expressed and associated with radiation response.

### Assessment of the false positive rate of the NCSRM

Additionally, in order to assess the false positive rate (statistical type I error, also called familywise error rate or FWER) we applied differential gene expression analysis using NCSRM between two technical replicates for all treatment groups. Because only two technical replicates were generated for each time point and treatment, we could not use the same approach to assess the technical variability for the BETR method, as it requires at least two replicates in each compared groups.

### Gene association network reconstruction from prior selected differentially expressed genes

Differentially expressed genes were subjected to gene association network reconstruction from time-course data using a regularized dynamic partial correlation method [54]. Pairwise relationships between genes over time were inferred based on a dynamic Bayesian network model with shrinkage estimation of covariance matrices as implemented in the GeneNet R-package available from CRAN [18]. Analyses were conducted with a posterior probability of 0.95 for each potential edge. Edge directions were not considered. In order to assess the complexity of the resulting networks, the density of each network was compared to the density of the Reactome functional interaction network [35, 55].

### Identification of important nodes in the network

Graph topological analyses based on centrality measures were applied in order to determine the importance of each node in the reconstructed association networks [56]. Three most commonly used centrality measures: degree, shortest path betweenness and closeness were combined into one cumulative centrality measure [34]. For each gene the three centrality values where ranked. The consensus centrality measure for each node was defined as the mean of the three independent centrality ranks. Combining centrality measures supports the identification of the nodes that are central themselves and also connected to direct central nodes, which demonstrates strategic positions for controlling the network.

### Pathway enrichment analysis

The Reactome pathway database was used to conduct the pathway enrichment analysis in order to further investigate the functions of the selected sets of differentially expressed genes [35]. Statistical significance of enriched pathways was determined by one-sided Fisher's exact test. The resulting p-values were adjusted for FDR using the Benjamini-Hochberg method. Pathways with FDR<0.05 were considered statistically significant and pathways were ranked according to ascending FDRs.

### Evaluation of NCSRM approach

Since we decided to use the set of genes that appeared to be differentially expressed we assessed the performance of the herein used NCSRM approach in comparison to the BETR approach implemented in the R/Bioconductor package betr [6]. BETR is a well-established algorithm that has been previously compared to limma, MB-statistic and EDGE methods and showed the best performance [6]. The results of spline and BETR methods were compared using the same initial microarray gene expression data set. The probabilities of each gene to be differentially expressed obtained with BETR method, were transformed to p-values as described in the original paper. Genes were considered significantly differentially expressed if the Benjamini-Hochberg adjusted p-value was lower than 0.05 (FDR<0.05). This transformation allowed us to compare the outcomes of both methods based on the FDR values for differential expression. The resulting differentially expressed genes using BETR were analyzed and subjected to network reconstruction as described above for the differentially expressed genes obtained using NCSRM. Outcomes of both obtained association networks were compared to each other and to the *a priori* known biological network provided by the Reactome database [35].

### Evaluation of reconstructed gene association networks

In order to assess the quality of the *de novo* reconstructed gene association networks (GANs), we developed a novel method that compares the interactions in the reconstructed network to the experimentally validated interactions present in the Reactome interaction network. For this purpose we used the Reactome reference network, consisting of protein-protein interaction pairs stored in the Reactome database (http://www.reactome.org/pages/download-data/). For the comparison, sub-networks of reconstructed networks consisting only of genes overlapping with the Reactome network were built. The number of common edges between these two sub-networks was determined and referred to the total number of edges in the reconstructed network (percentage of common edges in the reconstructed network). Further, a permutation test was performed to assess whether the number of common edges in the reconstructed network was significantly higher than in randomized networks with the same genes. Random networks were generated by permutation of the node names in the network, while preserving the reconstructed sub-network topology. After each permutation (n = 1000) the number of common edges with the reference Reactome sub-network was determined. The reconstructed network was considered significantly better than random, if more than 90% of the random sub-networks contained lower numbers of edges common with the Reactome network than the reconstructed sub-network (p-value < 0.1). All networks reconstructed with the genes determined as differentially expressed from the herein presented spline regression method and the BETR method were evaluated.

## Supporting Information

S1 File Reconstructed gene association networks. All obtained gene association networks are provided as R-objects of type igraph.
(RDATA)

S1 Table Lists of differentially expressed genes. Table includes differentially expressed genes identified by spline regression and BETR methods. Additionally, a list of overlapping differentially expressed genes between both methods is included.
(XLSX)

S2 Table Lists of significantly enriched pathways using differentially expressed genes identified by spline regression method. Four lists of significantly enriched pathways correspond to each used treatment condition. Lists include total numbers of known genes in the pathways, numbers of differentially expressed genes that belong to a single pathway (matches), percentages of differentially expressed genes in comparison to the total number of know genes in the pathway (% match), p-values, FDRs and names of pathways related differentially expressed genes.
(XLSX)

S3 Table Lists of 5% of most important genes identified by centrality measures. Lists of 5% highest ranked genes from the reconstructed gene association networks using spline regression and BETR methods. Overlap represents common most important genes identified in networks from compared methods.
(XLSX)

S4 Table Lists of pathways after mapping of 5% highest ranked genes from the reconstructed gene association networks. Lists include names of pathways together with names of mapped most important genes.
(XLSX)

S5 Table Significantly enriched senescence associated pathways with corresponding differentially expressed genes. Table presents the names of significantly enriched (FDR<0.05) senescence associated pathways with corresponding differentially expressed genes for all treatment conditions.
(XLSX)
